# The psychological burden of care: mental health and attrition risk among healthcare workers in the Czech Republic

**DOI:** 10.1192/j.eurpsy.2026.10362

**Published:** 2026-07-31

**Authors:** M. Janoušková, D. James Seblova, HEROES working group

**Affiliations:** Department of Public Health, Second Faculty of Medicine, Charles University, Prague, Czech Republic

## Abstract

**Introduction:**

High turnover and mental health problems among healthcare workers (HCWs) pose a serious threat to healthcare system stability, workforce sustainability, and the quality of patient care, with risks further amplified by the COVID-19 pandemic.

**Objectives:**

We aimed to examine how baseline and changing mental health indicators, as well as protective and workplace factors, predict turnover intention among Czech HCWs.

**Methods:**

We analysed data from the HEROES-CZ cohort. A longitudinal subsample of 668 HCWs was assessed at three timepoints, while a larger cross-sectional sample included 1,149 respondents in 2022. Depression (PHQ-9), psychological distress (GHQ-12), burnout, anxiety, and post-traumatic stress were examined as predictors of turnover intention. Logistic and multinomial regression models tested associations, adjusting for sociodemographics, occupation, physical illness, COVID-19 exposure, workplace trust, stigma, aggression, colleague support, and resilience.

**Results:**

In the cross-sectional sample, 37.6% of HCWs reported considering leaving healthcare. Women, younger respondents, and nurses were overrepresented, and this group had higher all measured mental health variables. Lower resilience, reduced colleague support, workplace stigma, and exposure to aggression were also associated with turnover intention. In the longitudinal analysis, higher baseline depression and distress predicted subsequent turnover intention, while changes over time did not. Older age was protective, whereas nurses reported higher turnover intention. Moderate workplace trust was linked to increased odds of turnover, while resilience protected against frequent turnover intention.

**Conclusions:**

Poor baseline mental health is a robust predictor of turnover intention, underscoring the importance of early identification and intervention. Resilience appears protective, and workplace trust, support, and stigma reduction are promising targets for retention strategies. Future longitudinal research should further clarify causal pathways.

**Disclosure of Interest:**

None Declared

